# Molecular Mapping of QTLs for Heat Tolerance in Chickpea

**DOI:** 10.3390/ijms19082166

**Published:** 2018-07-25

**Authors:** Pronob J. Paul, Srinivasan Samineni, Mahendar Thudi, Sobhan B. Sajja, Abhishek Rathore, Roma R. Das, Aamir W. Khan, Sushil K. Chaturvedi, Gera Roopa Lavanya, Rajeev. K. Varshney, Pooran M. Gaur

**Affiliations:** 1 International Crops Research Institute for the Semi-Arid Tropics (ICRISAT), Patancheru Hyderabad 502324, India; pronobjpaul@gmail.com (P.J.P.); s.srinivasan@cgiar.org (S.S.); t.mahendar@cgiar.org (M.T.); S.Sobhan@cgiar.org (S.B.S.); a.rathore@cgiar.org (A.R.); r.das@cgiar.org (R.R.D.); A.khan@cgiar.org (A.W.K.); r.k.varshney@cgiar.org (R.K.V.); 2 Department of Genetics and Plant Breeding, Sam Higginbottom University of Agriculture, Technology and Sciences (SHUATS), Allahabad 211007, India; lavanya.roopa@gmail.com; 3 ICAR-Indian Institute of Pulses Research (ICAR-IIPR), Kanpur 208024, India; sushilk.chaturvedi@gmail.com; 4 The UWA Institute of Agriculture, University of Western Australia, Perth, WA 6009, Australia

**Keywords:** abiotic stress, *Cicer arietinum*, candidate genes, genetics, heat-stress, molecular breeding

## Abstract

Chickpea (*Cicer arietinum* L.), a cool-season legume, is increasingly affected by heat-stress at reproductive stage due to changes in global climatic conditions and cropping systems. Identifying quantitative trait loci (QTLs) for heat tolerance may facilitate breeding for heat tolerant varieties. The present study was aimed at identifying QTLs associated with heat tolerance in chickpea using 292 F_8-9_ recombinant inbred lines (RILs) developed from the cross ICC 4567 (heat sensitive) × ICC 15614 (heat tolerant). Phenotyping of RILs was undertaken for two heat-stress (late sown) and one non-stress (normal sown) environments. A genetic map spanning 529.11 cM and comprising 271 genotyping by sequencing (GBS) based single nucleotide polymorphism (SNP) markers was constructed. Composite interval mapping (CIM) analysis revealed two consistent genomic regions harbouring four QTLs each on CaLG05 and CaLG06. Four major QTLs for number of filled pods per plot (FPod), total number of seeds per plot (TS), grain yield per plot (GY) and % pod setting (%PodSet), located in the CaLG05 genomic region, were found to have cumulative phenotypic variation of above 50%. Nineteen pairs of epistatic QTLs showed significant epistatic effect, and non-significant QTL × environment interaction effect, except for harvest index (HI) and biomass (BM). A total of 25 putative candidate genes for heat-stress were identified in the two major genomic regions. This is the first report on QTLs for heat-stress response in chickpea. The markers linked to the above mentioned four major QTLs can facilitate marker-assisted breeding for heat tolerance in chickpea.

## 1. Introduction

In recent years, the adverse impact of climate change on agriculture is well recognized all over the globe. The ever-increasing day and night temperature is going to affect the production of crops, especially those grown in the winter [1]. In this context, heat-stress due to rise in temperatures remains a challenge in developing crop varieties that are adaptive to changing climatic conditions.

Chickpea is a nutrient-rich grain legume crop cultivated in arid and semi-arid regions. The chickpea grain is an excellent source of proteins along with a wide range of essential amino acids and vitamins. In the fight against hidden hunger all over the globe, the role of legumes such as chickpea is indispensable. Grown in over 60 countries and traded in over 190 countries, chickpea is the second most consumed pulse crop in the world after common bean [2]. Due to global warming, several noticeable changes occurred in the cropping system and intensity in the recent past. These are delaying the cultivation of chickpea to relatively hot conditions [1]. Generally, the crop faces heat-stress during reproductive phase under late sown condition in the tropical and semi-arid regions [3]. Reports state that the exposure to temperature, 35 °C and above, even for a few days, during reproductive phase has a negative impact on optimum yield in chickpea [4,5]. Unlike drought and other abiotic stresses, until recently, the importance of breeding for heat-stress conditions in chickpea has not been realized [1]. 

Grain yield under heat-stress is considered to be one of the important criteria for assessing heat tolerance in chickpea [3,4,5]. However, chickpea yield is known to be highly influenced by environments [6]. Due to genotype by environment (G × E) interaction, breeding for heat tolerance through conventional breeding approaches based on yield parameter sometimes limits selection for heat-stress tolerance in chickpea. 

In recent years, progress has been made in genomics-enabled trait dissection in several crop plants, including chickpea. Several studies have been carried out earlier to identify the quantitative trait loci (QTLs) for tolerance to various biotic stresses [7,8], and abiotic stresses like drought tolerance [9], and salinity tolerance [10,11,12] in chickpea. Moreover, genomic regions associated with heat tolerance have been reported in several crops, including wheat, rice, maize, barley, potato, tomato, cowpea, azuki bean, brassica [13]. Pod setting (seed set) and grain yield have been used as proxy traits to detect QTLs for heat tolerance in different crops [14,15,16,17,18]. Similarly, in chickpea, the number of filled pods, total number of seeds, biomass, and harvest index were found to be significantly associated with heat tolerance [3,19]. However, to date, QTLs for heat tolerance have not been reported in chickpea. 

In this study, genotyping by sequencing (GBS)-based single nucleotide polymorphism markers were used to identify key genomic regions responsible for heat tolerance. In addition, putative candidate genes for heat tolerance in these genomic regions were identified using the available chickpea genome sequence information [20]. 

## 2. Results

### 2.1. Response of Parents and Recombinant Inbred Lines (RILs) under Heat-Stress and Non-Stress Environments

**Figure 1:**
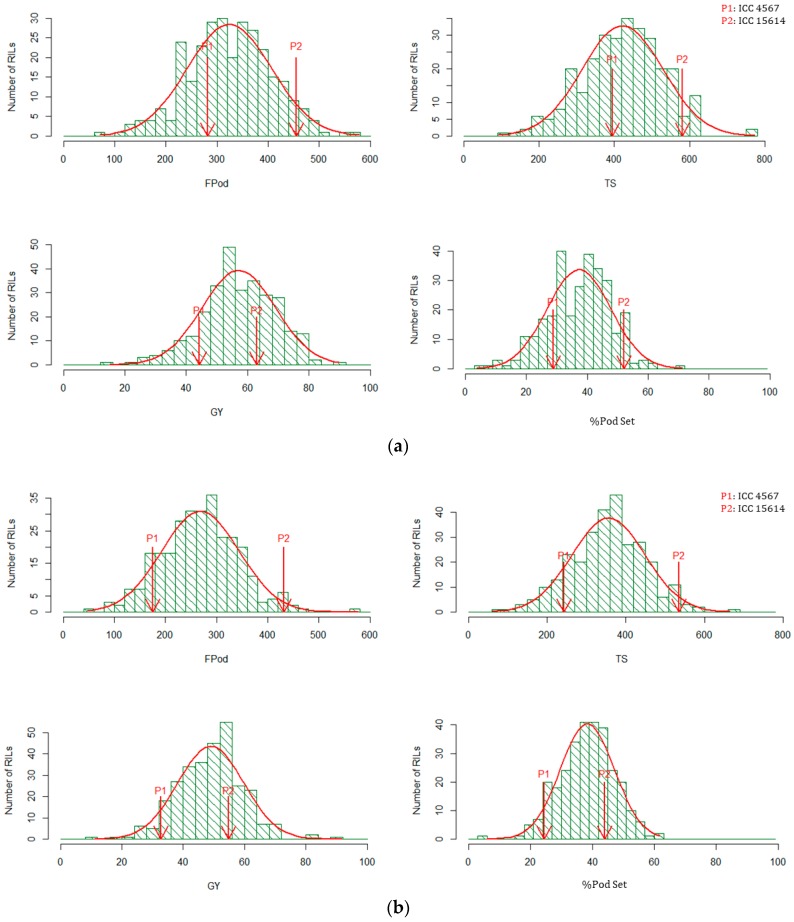
(**a**) Frequency distribution of Number of Filled Pods per Plot (FPod), Total Number of Seeds per Plot (TS), Grain Yield per Plot (GY, g), and Percent Pod Setting (%PodSet) in RIL population (ICC 4567 × ICC 15614). P1 is heat sensitive parent ICC 4567 and P2 is heat tolerant parent ICC 15614. The left portion of the P1 on the *X*-axis indicates the negative transgressive segregants, conversely, the right portion of the P2 on the *X*-axis indicates the positive transgressive segregants in heat-stress environment, 2013; (**b**) Frequency distribution of Number of Filled Pods per Plot (FPod), Total Number of Seeds per Plot (TS), Grain Yield per Plot (GY, g), and Percent Pod Setting (%PodSet) in RIL population (ICC 4567 × ICC 15614). P1 is heat sensitive parent ICC 4567 and P2 is heat tolerant parent ICC 15614. The left portion of the P1 on the *X*-axis indicates the negative transgressive segregants, conversely, the right portion of the P2 on the *X*-axis indicates the positive transgressive segregants in heat-stress environment, 2014.

The descriptive analysis of parents and RILs are presented in Table 1. Predicted means for all the traits in parents differed significantly in both heat-stress environments, except biomass per plot (BM). In the non-stress environment, predicted means for grain yield per plot (GY), BM, harvest index (HI) and %PodSet were non-significant between parents, while filled pods per plot (FPod) and total number of seeds per plot (TS) were significant. The range of variation in all the traits was high in stress environments (Table 1). The combined analysis of variance (ANOVA) for both the stress environments revealed that significant variation existed in RILs for all the traits measured, except BM, whereas under non-stress environment relatively low genetic variability was observed. Transgressive segregants in both directions were observed for several traits in the RIL population (Figure 1a,b).

The potential use of a trait in a breeding program relies on the heritability of that trait. Under both the heat-stress conditions, the heritability of all the traits was high (72.0–90.7%), except BM in summer 2014 (49.8%). Whereas, under non-stress environment the heritability of the traits was moderate (47.6–66.0%) (Table 1).

### 2.2. Relationship between Yield and Yield Determining Traits

Heat tolerance is a complex trait and can be estimated indirectly through yield and yield contributing traits under heat-stress. All the traits- visual score (VS), FPod, TS, BM and %PodSet were positively correlated with yield (*r* = 0.51 **–0.90 **) under both the heat-stress environments and pooled over analysis except HI (*r* = 0.32 **) under heat-stress environment of 2013 (Table 2). In addition, VS had positive association with FPod (*r =* 0.68 **–0.80 **) and TS (*r* = 0.67 **–0.79 **). Likewise, %PodSet was found to have a strong positive correlation with FPod (*r* = 0.59 **–0.77 **) and TS (*r* = 0.60 **–0.78 **) under both the heat-stress environments as well as in pooled analysis (Table 2). In contrast, under non-stress environment, the correlation with yield was low for %PodSet (*r* = 0.17 **) and HI (*r* = 0.33 **), and high for other traits (*r* = 0.63 **–0.91 **) (Table 2). Regression analysis between the traits and yield revealed that all the traits exhibited medium to high variation for yield (25% to 81%) in both stress environments as well as pooled over years (Figure S3a–c). In non-stress environment, %PodSet had low contribution (3%) whereas BM was found to have high variation for yield (82%) (Figure S3d). A significant correlation between the yield and yield contributing traits under heat-stress environment indicated that these traits can be used in direct or indirect selection for improving heat tolerance in chickpea.

### 2.3. Sequencing Data and SNP Discovery

The parents of the mapping population (ICC 4567 × ICC 15614) were sequenced at higher depth (5× coverage), and a total of 19.63 million reads containing 1.70 Gb for ICC 4567, and 15.79 million reads containing 1.37 Gb for ICC 15614, were generated. In addition, 3333.41 million reads containing 289.70 Gb were generated from 292 RILs. The number of reads generated varied from 6.86 million (RIL099) to 20.66 million (RIL112) with an average of 11.42 million per line. The single nucleotide polymorphisms (SNPs), identified using the software SOAP, were analyzed to remove heterozygous SNPs in the parents, and a set of 396 SNPs were identified across 292 RILs. The sequence details of all SNPs have been provided in Table S1a,b.

### 2.4. Genetic Linkage Map and Marker Distribution

The 396 polymorphic SNPs obtained from GBS were used for genetic map construction. The genetic linkage map covered 529.11 cM of the chickpea genome with an average interval of 1.95 cM between markers (Table S2 and Figure S1). The highest number of markers was in CaLG04 (57), while the lowest number of markers was in CaLG08 (10) (Figure S1). CaLG08 showed the highest marker density with 1.78 markers per cM on average. The lowest marker density was observed for CaLG02, which had 0.29 markers per cM on average. Overall, the map had on average 0.51 markers per cM (Table S2).

### 2.5. QTL Analysis

#### 2.5.1. Genomic Region on CaLG05

**Figure 2:**
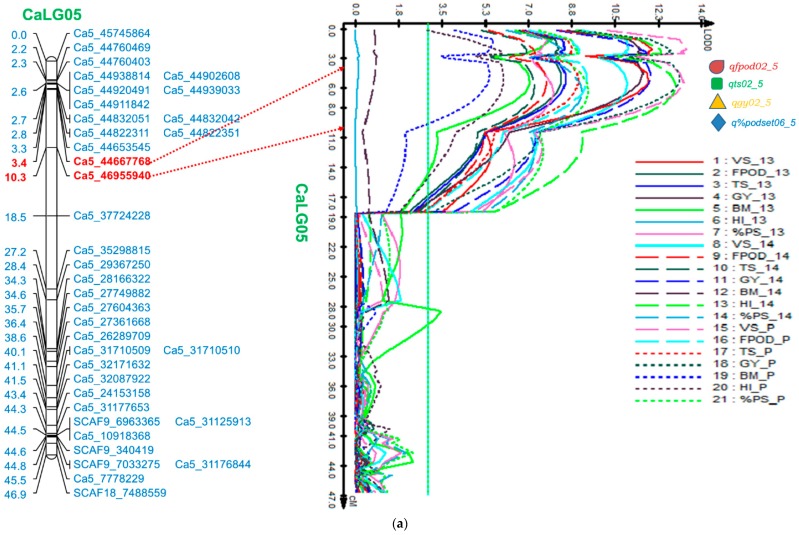

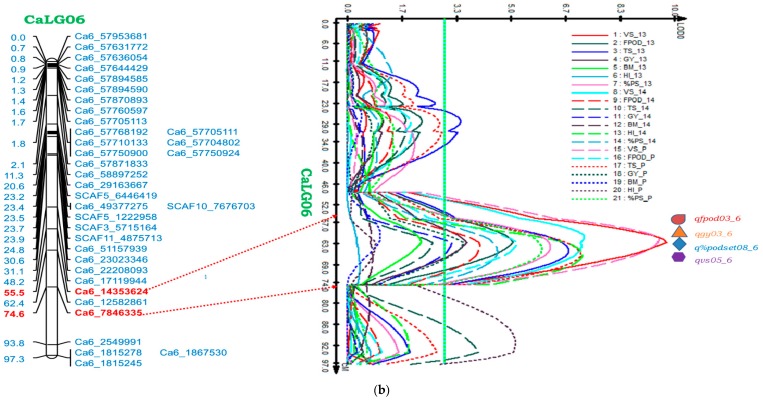
(**a**) Likelihood of odds ratio (LOD) curves obtained by composite interval mapping for quantitative trait loci (QTL) mapped over two heat-stress environments, 2013, 2014 and their pooled years together. Four major QTLs-*qfpod02_5*, *qts02_5*, *qgy02_5*, *q% podset06_5* of the four traits-Number of Filled Pods per Plot (FPod), Total Number of Seeds per Plot (TS), Grain Yield per Plot (GY) and Percent Pod Setting (%PodSet) in the genomic region on CaLG05 flanked by markers Ca5_44667768 and Ca5_46955940. The vertical lines indicate the threshold LOD value (2.5) determining significant QTL; (**b**) Likelihood of odds ratio (LOD) curves obtained by composite interval mapping for quantitative trait loci (QTL) mapped over two heat-stress environments, 2013, 2014 and their pooled years together. Four QTLs, *qfpod03_6*, *qgy03_6*, *q% podset08_6*, *qvs05_6* for the traits Number of Filled Pods per Plot (FPod), Grain Yield per Plot (GY), Percent Pod Setting (%PodSet) and visual score on podding behaviour (VS) in the genomic region on CaLG06 with the marker interval Ca6_14353624-Ca6_7846335, in the RIL mapping population of ICC 4567 × ICC 15614. The vertical lines indicating the threshold LOD value (2.5) determining significant QTL.

A promising genomic region harbouring major QTLs for four traits—FPod, TS, GY, and %PodSet flanked by markers Ca5_44667768 and Ca5_46955940—was identified on CaLG05 (Table 3). The four QTLs—*qfpod02_5*, *qts02_5*, *qgy02_5*, and *q%podset06_5*—were found in both the stress environments spanning 6.9 cM (corresponding to ~2.28 Mb on physical map) (Figure 2a). The phenotypic variation for GY-QTL (*qgy02_5*) was 16.04% (LOD 11.69) and 16.56% (LOD 12.00) in heat-stress environments I (2013) and II (2014), respectively. QTLs for FPod—*qfpod02_5* in this genomic region demonstrated phenotypic variation of 11.57% (LOD 8.37) and 12.03% (LOD 7.79), respectively, in the consecutive stress environments (Table 3). Similarly, QTLs for the TS *qts02_5* in heat-stress environments I (2013) and II (2014) explained phenotypic variation of 12.0% (LOD 8.54) and 10.0% (LOD 7.30). The QTL for %PodSet (*q% podset06_5*), which has been considered as an important selection criterion for heat tolerance in chickpea, had a phenotypic variation of 11.51% (LOD 8.04) and 13.30% (LOD 9.20) in the heat-stress environments of 2013 and 2014, respectively (Table 3). 

All the major QTLs present in the genomic region of CaLG05 were found to exist in the pooled analysis for the two stress environments (Table 3). In CaLG05, two major QTLs for VS and HI were found explaining 15.1% (LOD 11.1) and 18.5% (LOD 13.0) of phenotypic variation, under the heat-stress environment (2014), respectively (Table S3). In contrast, during the stress environment in 2013, one major QTL for VS was found close to the genomic region on CaLG05 with a phenotypic variation of 13.88% (LOD 12.05) (Table S3). Through single marker analysis (SMA), Ca5_44667768 was co-segregated with the four major QTLs in this genomic region.

#### 2.5.2. Genomic Region on CaLG06 

A second genomic region, harbouring QTLs for four important traits in this study, was identified having the marker interval Ca6_14353624—Ca6_7846335 (Table 3 and Figure 2b). The QTLs for FPod (*qfpod03_6*), GY (*qgy03_6*), %PodSet (*q% podset08_6*), and VS (*qvs05_6*) spanned a genetic length of 19.14 cM (~6.50 Mb on physical map) in CaLG06. The range of phenotypic variation shown by various traits in this genomic region was from 3.92 to 11.07% (Table 3).

#### 2.5.3. QTLs Identified on Other LGs

In the present work, a total of 13 QTLs were identified consistently across two heat-stress environments showing both major and minor effects for various traits measured. Apart from the QTLs identified in CaLG05 and CaLG06, a QTL for GY (*qgy01_1*) was found in the same position (40.0 cM) demonstrating 7.33% and 10% of phenotypic variation in the first and second year, respectively, on CaLG01 (Table S4).

On CaLG02, QTL for FPod (*qfpod01_2*) occurred at the same position (65.81 cM) in consecutive years with a phenotypic variation of 4.9% (LOD 3.38) and 5.8% (LOD 4.0). Similarly, QTL for TS (*qts01_2*) was found explaining 5.6% and 8.1% phenotypic variation under heat-stress environments (2013 and 2014), respectively. A major QTL (*q%podset03_4*) with phenotypic variation 12.5% (LOD 4.72) for %PodSet in 2013 was also observed in 2014 with 7.8% phenotypic variation and LOD value of 3.6 with same marker interval (Ca4_13699195-Ca4_7818876) on CaLG04 (Table S4).

#### 2.5.4. Mapping of Epistatic QTLs (E-QTLs)

Epistatic interaction analysis revealed that 19 QTL pairs were involved in the epistatic interactions covering seven LGs (Table 4). A significant effect was observed for all the epistatic interactions. However, no significant interaction between epistasis and environment was observed, except for the trait biomass (BM).

Two epistatic QTL pairs for VS were found to have loci distributed on four different LGs accounting for 3.43% phenotypic variation. In the case of FPod, two QTLs were found to be interacting in the same LG, CaLG02. Another QTL pair was found for FPod to interact with each other in two different LGs (Table 4). These two epistatic QTL pairs for FPod together explained a phenotypic variation of 2.94%. 

The highest number of epistatic QTL pairs (nine pairs) were detected for TS in this population and have contributed up to 12.38%. The epistatic interaction for TS was found in all the linkage groups, except CaLG03 and CaLG07. One QTL interaction pair was detected for GY interacting from CaLG01 to the locus on CaLG02 with a phenotypic variation 0.83% (Table 4 and Figure S2). Similarly, in the case of %PodSet, four epistatic QTL pairs were found to interact with each other in three linkage groups CaLG01, CaLG03, and CaLG04 showing a phenotypic variation of 5.79%. 

In addition, an interaction between non-QTL, and additive and additive × environment-QTL was found in the case of BM, which showed 1.22% phenotypic variation. Concurrently, five loci (loci located at 10.1 cM and 26.4 cM in CaLG01, 2.2 cM and 75.6 cM in CaLG04, and at 44.5 cM in CaLG05) were observed to have interaction simultaneously with several other loci affecting the expression of the particular trait. Two loci (*eqts2_1/eqpodset2_1* in CaLG01 and *neqfpod4_5/neqts9_5* in CaLG05) controlling two or three different traits were also interacted with other loci (Table 4).

## 3. Discussion

### 3.1. Phenotypic Evaluation of RILs and Parents in Field Condition

Sowing during the month of February proved to be an ideal condition to expose chickpea crop to heat-stress and selecting heat tolerance lines in earlier studies under field conditions at ICRISAT, Patancheru, India [19,21]. A recent study on chickpea reported 34 °C as the threshold temperature for pod setting and also observed that at 35 °C, pod set was reduced by 50% in chickpea genotypes [19]. The average maximum temperatures (37.5 °C and 36.7 °C in summer 2013 and summer 2014, respectively) in both the heat-stress environments found were ideal for phenotyping RIL population. An average maximum temperature of 29.4 °C was recorded in non-stress environment, which was considered as control for this study. This temperature was ideal for sowing in the non-stress environment for the timely sown crop [22]. 

The frequency distribution of measured traits showed the characteristics of continuous variation (Figure 1a,b). Paliwal et al. (2012) [23] in RILs of wheat and Buu et al. (2014) [24] in BC_2_F_2_ population in rice, reported several transgressive segregants for heat tolerance. Similarly, in this present study, transgressive segregants in both directions were observed, indicating that both parents have contributed alleles for heat tolerance in the RILs (Figure 1a,b). A significant variation found among the RILs for all the traits indicate the presence of genetic diversity in the selected parents for the selected traits under heat-stress condition. Parents differed significantly for all the traits in both the heat-stress environments, except biomass (BM). 

High heritability (H^2^) values were observed for all the traits measured under both the heat-stress environments, except for biomass in summer 2014, which indicates that there is a high probability of achieving the same kind of results if the trial is repeated under similar growing conditions. 

Yield under high temperatures is the key objective for heat tolerance breeding in chickpea. Traits such as FPod, %PodSet and TS contributing to increased yield under high-temperature stress can be treated as a proxy for heat tolerance. The presence of significant correlations between yield and other traits in heat-stress environments indicated that these traits can be used as selection criteria for heat tolerance. 

FPod and TS had a strong correlation with yield (88 to 90%) under both the stress environments. Such high correlation of these traits toward yield was reported earlier in chickpea under abiotic stress [10,11]. In addition, VS and %PodSet was also found to have good correlation (50 to 79%) with yield. However, BM and HI showed large difference in correlation with yield in both the heat-stress conditions. Positive and strong association of the four traits-FPod, TS, VS and %PodSet with grain yield revealed the importance of these traits in determining yield under heat-stress environment. Hence, detecting QTLs of these traits under stress would be helpful in heat tolerance programme.

### 3.2. QTL Mapping for Heat Tolerance

The genomic region in CaLG05 harbours QTLs for FPod, TS, GY, and %PodSet, which were reportedly associated with heat tolerance in chickpea [3,19]. Interestingly, the positions of the QTLs (*qts02_5*, *qgy02_5*, *q% podset06_5*) for TS, GY, and %PodSet were identified in the same position over the years, which strongly confirm the QTLs in these positions. 

The presence of four major co-localized QTLs (*qfpod02_5*, *qts02_5*, *qgy02_5*, *and q% podset06_5*) suggests tight linkage or the phenomenon of pleiotropy and the phenotypic correlations between these traits were highly significant in both the stress environments. Moreover, the tolerant parent ICC 15614 is contributing the desirable alleles for all the QTLs found in the two genomic regions in CaLG05 and CaLG06. 

Identification of QTLs at the same positions in both the heat-stress environments indicate their possible practical utility in breeding for heat-stress tolerance in subsequent studies [25]. Several co-localized QTLs for various traits were found which could possibly due to pleiotropy or tightly linked QTLs. Fine mapping of the target genomic region will further help in resolving the issues of pleiotropy and tight linkage. The incorporation of a higher number of markers into the existing genetic map can further narrow down the genomic regions identified.

QTLs for traits such as FPod, TS, and GY were not expressed under non-stress condition, confirming the fact that these QTLs were only expressed under high-temperature condition. Two major QTLs for HI were identified in CaLG01 and CaLG04 explaining the phenotypic variation of 12.03% (LOD 8.8) and 12.53% (LOD 7.9), respectively. In addition, three minor QTLs including one for HI and two for %PodSet were found in different LGs. The fewer number of detected QTLs and their unique positions in the non-stress environment is a strong evidence that there is no correspondence between QTLs found in non-stress with the QTLs found in heat-stress environment. This phenomenon proves the fact that those QTLs identified in heat-stress condition were independent and exclusive for heat tolerance.

### 3.3. Epistatic QTLs for Heat Tolerance

Epistatic interaction is one of the key factors controlling the expression of a complex trait. The epistatic interaction analysis of QTLs provides a more comprehensive knowledge of the QTLs and their genetic behaviour underlying the trait [26,27]. 

In the current study, 19 pairs of digenic epistatic QTLs were found to be associated with the six traits: VS, FPod, TS, GY, BM, and %PodSet. Maximum number epistatic QTLs loci were observed for TS (nine), followed by %PodSet (four). In this study, some loci such as *eqts2_1/eqpodset2_1*, *eqts2_1/eqpodset2_1*, *eqpodset2_1/eqts2_1*, *neqts9_5/neqfpod4_5*, *neqfpod4_5/neqts9_5* were simultaneously controlling more than one trait indicating the pleiotropy nature of the traits. 

Four categories of epistatic interaction were found in this study such as, additive × additive, additive × non-QTL, non-QTL × non-QTL, and additive × (additive-environment) × non-QTL interaction. FPod and VS showed two epistatic interactions each. Out of two epistatic interactions, one additive × additive epistatic interaction was found for both FPod and VS. 

For GY, one additive × additive QTL epistatic interaction was found. For TS, five additive × additive QTL epistatic interactions, three non-QTL × non-QTL interaction and one additive × non-QTL interactions were observed. Similarly, two additive × additive QTL interactions, one non-QTL × non-QTL interaction and one additive × non-QTL interaction were observed for %PodSet. All the epistatic interactions were found to be significant.

The additive effects were found in both directions for all the traits. Nine interactions had negative additive effects, meaning that recombinant allele combinations could increase the particular trait value. Similarly, ten epistatic QTL interactions having positive additive effects, indicating parental allele combinations, would help to improve the trait [28].

Presence of epistatic interactions for a given trait will make the selection difficult. Interestingly, all major QTLs had no epistatic interaction and this will increase the heritability of the trait and make the selection easy. 

### 3.4. Putative Candidate Genes for Heat Tolerance 

Recent progress in functional genomics facilitates the elucidation of the important role of candidate genes for expression of tolerance against abiotic stress in plants [29,30,31]. In the present study, mining of the candidate genes for heat tolerance revealed 236 genes in 2.28 Mb (44.6–46.9 Mb) region in CaLG05 and 550 genes in 6.50 Mb (7.85–14.35 Mb) in CaLG06 (Tables S5 and S6). Based on functional categorization, many genes were found to be associated with biological processes (168 genes in CaLG05 and 365 genes in CaLG06) in the two genomic regions. 

Gene ontology classification revealed a total of 25 putative candidate genes (11 in CaLG05 and 14 in CaLG06) known to function, directly or indirectly, as heat-stress response genes in several plant species (Table S9a,b). Of the 25 candidate genes, five genes encode protein like farnesylated protein 6 (AtFP6), ethylene-responsive transcription factor ERF114, ethylene-responsive transcription factor CRF4, F-box protein SKP2B, and ethylene-responsive transcription factor RAP2-11. These genes were identified to have key roles in heat acclimation and growth of plants under severe heat-stress condition. Many transcription factors, enzyme, and stress responsive element binding factors responsible for heat tolerance in various plant species were reported earlier [32]. Furthermore, various heat shock proteins (HSPs), ethylene forming enzymes (EFEs), and ethylene-responsive element factors (ERFs) were found to be candidate genes for heat tolerance in soybean and cowpea, two of the plant species closest to chickpea [32]. 

The role of various heat shock proteins and heat-stress transcription factors has been widely accepted and reported in different crops [33]. The role of HSP90 transcription factors under heat-stress conditions was also reported in chickpea [34]. Five putative genes were identified in the two examined genomic regions, encoding for either heat shock proteins or heat shock transcription factors contributing for thermo-tolerance. 

Oxidative stress can occur in parallel with heat-stress through the formation of reactive oxygen species (ROS) [35]. Three putative candidate genes were also observed in this study to have a role in defying oxidative stress and recovering plants from heat-stress damage. These genes encode different types of proteins like protein tansparent testa glabra 1, peroxidase 52, and zinc finger protein CONSTANS-LIKE 5. In addition, certain signalling molecules like ethylene, abscisic acid (ABA), and salicylic acid are among a few to have a significant role in the development of heat tolerance [36]. In this study, a few genes—MYB44, AKH3, and RAN1—were found to involve with these signalling molecules through upregulation process to mitigate the heat-stress. Being a preliminary study, evaluation of these putative candidate gene-functions in chickpea through fine mapping and gene expression study is necessary to use them for further study.

## 4. Materials and Methods

### 4.1. Plant Material and Treatment Condition

A mapping population of 292 RILs developed from a cross between a heat sensitive parent ICC 4567 and a heat tolerant parent ICC 15614 was used for the study. Field experiments were carried out at ICRISAT, Patancheru, India (17°30′ N; 78°16′ E; altitude 549 m) on a vertisol soil. The F8-9 RIL population was evaluated under two heat-stress environments (in summer, February–May 2013 and February–May 2014) and in one non-heat-stress environment (in winter, November–February 2013). 

In all the environments, the field was solarized using polythene mulch during the preceding summer to sanitize the field, especially to avoid incidence of root diseases. Sowing was done on the ridges using ridge and furrow method with inter- and intra-row spacing of 60 × 10 cm. Each plot consisted of a 2 m long row. Need-based insecticide sprays were provided to control pod borer (*Helicoverpa armigera*) and the experimental plots were kept weed-free through manual weeding. Before sowing, seeds were treated with the mixture of fungicides 0.5% Benlate^®^ (E.I. DuPont India Ltd., Gurgaon, India) + Thiram^®^ (Sudhama Chemicals Pvt., Ltd., Gujarat, India).

The experimental design was laid out in a 15 × 20 alpha lattice design with three replications. The sowing for the non-stress environment was done on the residual moisture in the last week of November 2013 and provided with essential irrigation. The planting was done in the first week of February for stress environments to expose the reproductive phase of RILs to heat-stress (>35 °C). The stress experiments were provided with irrigation to avoid the confounding effect of moisture stress during the heat screening.

**Figure 3:**
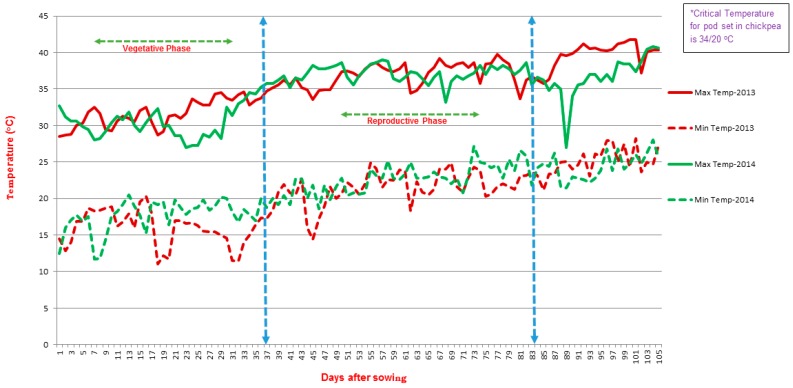
Daily maximum and minimum temperatures (°C) during the late sown crop growing period (stress season) in 2013 and 2014 (34/19 °C is the threshold temperature for the maximum and minimum temperatures for chickpea yield, respectively. The maximum day temperatures were 39.8 °C and 39.0 °C, and maximum night temperatures were 24.9 °C and 27.2 °C in heat-stress environments 2013, and 2014, respectively. Crop growing period was 2nd week of February to 3rd week of May).

In chickpea, a temperature higher than 35 °C during reproductive phase adversely affects growth, development, and yield [1,19]. The parents used for developing RIL population for this study showed significant variations at this temperature (35 °C and above) in an earlier study [19] (Devasirvatham et al., 2013). The mean daily day/night temperatures during the reproductive phase of RILs in heat-stress environment 2013 and heat-stress environment 2014 were 37.5/22.5 °C and 36.7/22.9 °C, respectively (Figure 3). Whereas under normal season (non-stress environment), the mean daily temperatures were 29.6/15.5 °C.

### 4.2. Variables Measured

Number of filled pods per plot (FPod), total number of seeds per plot (TS), grain yield per plot (GY, g), harvest index (HI, %), biomass (BM, g) and percent pod setting (%PodSet), were reportedly found to be associated with heat tolerance in chickpea [3,19]. These six traits along with visual score on podding behaviour (VS) were recorded in the RIL population. The data for FPod, TS, GY, BM, and HI were recorded from a half-meter (0.5 m) long continuous patch out of the 2-m plot. VS at maturity and %PodSet were recorded from the entire plot. For visual scoring, score-1 was considered most sensitive (least number of pod-bearing ability), whereas, score-5 was taken as the most tolerant (maximum number of pod-bearing ability) under heat-stress. In the non-stress environment, all RILs were assumed to behave more or less the same. Hence, no visual score data were recorded in this environment.

### 4.3. DNA Extraction, Genotyping, and SNP Calling

DNA from 292 RILs, along with the parents, was isolated from 15-day old seedlings following the high-throughput mini-DNA extraction method [37]. Genotyping was done using GBS approach [38]. The GBS libraries from the parental lines and RILs were prepared using ApeKI endonuclease (recognition site: G/CWCG) and were sequenced using the Illumina HiSeq 2000 platform (Illumina Inc, San Diego, CA, USA). The detailed procedure of genotyping approach was described by Jaganathan et al. (2015) [25]. 

For SNP calling the raw reads obtained were first de-bimultiplexed using sample barcodes, and adapter sequences were removed using a custom Perl script (Figure S5). The reads having more than 50% of low-quality base pairs (Phred < 5%) were discarded and filtered data were used for calling SNPs after due quality check (Q score > 20). The high-quality data from each sample were aligned to the draft genome sequence (CaGAv1.0) of chickpea [20] using SOAP [39]. After SNP calling, the polymorphic loci were determined by following the criteria defined in [25].

### 4.4. Linkage Map Construction, QTL Detection and Mining of Candidate Genes

By adopting a stringent selection criterion including the missing percentage, minor allele frequency, and percent heterozygosity, the final number of SNPs included in the analysis were 396. The selected panel of robust SNPs were used for construction of genetic maps. 

A linkage map was constructed with the 396 SNPs using JoinMap 4.1 [40]. Composite interval mapping in QTL Cartographer-V 2.5 [41] was employed to identify the QTLs responsible for heat tolerance with a forward and backward stepwise regression (threshold *p*-value < 0.05). A window size of 10 cM, along with a walking speed of 1.0 cM, and 1000 permutations for *p* < 0.05 were chosen for the QTL analysis. QTL × QTL and QTL × E interactions were estimated using the QTL Network version 2.0 (http://ibi.zju.edu.cn/software/qtlnetwork/) which is based on a mixed linear model. 

First-dimensional genome scan (with the option to map epistasis) and second-dimensional genome scan (to detect epistatic interactions with or without single-locus effect) were applied. A significance level of 0.05 with 1000 permutations, 1.0 cM walk speed, 10.0 cM testing window and filtration window size were employed for the epistatic QTL analysis. QTL was named with prefix “q” for main-effect QTL, “eq” for epistatic QTL and “neq” for non-QTL epistasis followed by the abbreviated trait name and the identity of the linkage group involved.

The identified markers along with the flanking sequences were mapped on the chickpea reference genome CaGAv1.0 [20]. The genes present within the physical locations of these markers were extracted from the genome features file and were searched against TrEMBL and Swiss-Prot databases. Further functional annotation was done using UniProtKB. The Gene Ontology annotations were categorized into three categories: biological processes (BP), molecular function (MF) and cellular components (CC).

### 4.5. Statistical Analyses

#### Analysis of Variance, Predicted Means (BLUP), Heritability, and Correlations

The analysis of variance (ANOVA) for the RIL population was performed using GenStat (17th Edition), for individual environments using mixed model analysis. For each trait and environment, the analysis was performed considering entry and block (nested within replication) as random effects and replication as fixed effect. 

To pool the data across environments, and to make the error variances homogeneous, individual variances were estimated and modelled for the error distribution using residual maximum likelihood (ReML) procedure. *Z* value and *F* value were calculated for random effects and fixed effects, respectively. For single and multi-environment, QTL mapping was performed using predicted means (BLUP-Best Linear Unbiased Prediction) [42]. 

Broad-sense heritability was estimated by following Falconer et al., 1996 [43] as

H^2^ = Vg/(Vg + Ve/nr);

and pooled broad-sense heritability was estimated by following Hill et al., 2012 [44] as

H^2^ = Vg/{(Vg) + (Vge/ne +Ve/(ne × nr))}

Whereas, H^2^ is broad-sense heritability, Vg is genotypic variance, Vge is G × E interaction variance, Ve is residual variance, ne is number of environments, and nr is number of replications. Pearson correlation analysis and linear regressions were fitted using Microsoft Excel 2016 (Microsoft Corp., 1985, Redmond, WA, USA).

## 5. Conclusions

The present study identified two potential genomic regions harbouring major QTLs for several heat responsive traits that are directly related to heat tolerance in chickpea. The two regions consistently appeared at the same map position across two years. Epistatic effects were not observed for major QTLs and no QTL × E interaction in the CaLG05 region. The results laid a foundation in understanding heat tolerance and increases the confidence of breeders to proceed with early generation selection for heat tolerance through marker-assisted breeding. In addition, the candidate genes identified in the two genomic regions further help to understand the mechanism of heat tolerance.

## Figures and Tables

**Table 1:** Summary statistics and heritability (H^2^) values for the measured traits of 292 RILs in non-stress and heat-stress environments.

Trait	Visual Score	Filled Pods Plot^−1^	Total No. of Seeds Plot^−1^	Grain Yield Plot^−1^ (g)	Biomass Plot^−1^ (g)	Harvest Index	Percent PodSet (%)
**Non-stress Environment, 2013**							
ICC 4567 (heat sensitive)	-	406.8	429.2	76.0	144.8	52.1	67.7
ICC 15614 (heat tolerant)	-	538.7	553.0	70.2	132.3	53.9	75.6
Contrast analysis between parents	-	−131.9 *	−123.9 *	5.8 ^ns^	12.5 ^ns^	−1.9 ^ns^	−7.9 ^ns^
Mean of RILs	-	459.0	486.3	73.5	139.7	53.0	68.8
Range of RILs	-	360.8–580.1	378.3–604.7	57.6–93.3	118.1–165.2	45.5–59.2	48.1–84.2
Heritability (%)	-	62.1	60.5	57.6	47.6	63.4	66.0
**Heat-stress environment, 2013**							
ICC 4567 (heat sensitive)	2	281.3	395.1	44.3	147.6	34.2	28.8
ICC 15614 (heat tolerant)	5	455.6	580.7	62.9	125.9	50.6	52.0
Contrast analysis between parents	−0.5 *	−174.3 *	−185.6 *	−18.6 *	21.7 ^ns^	−16.3 *	−23.1 *
Mean of RILs	3.0	323.9	421.3	57.1	114.6	50.6	37.3
Range of RILs	(1–5)	70.5–578.3	91.9–772.4	14.9–89.8	32.9–185.6	34.5–69.1	3.7–71.3
Heritability (%)	79.8	86.9	86.3	82.2	83.2	72.0	90.7
**Heat-stress environment, 2014**							
ICC 4567 (heat sensitive)	2	175.3	242.0	32.6	123.2	23.9	24.4
ICC 15614 (heat tolerant)	5	431.2	534.9	54.8	111.6	52.0	43.9
Contrast analysis between parents	−0.6 *	−255.9 *	−292.9 *	−22.1 *	11.7 ^ns^	−28.2 *	−19.6 *
Mean of RILs	3.0	268.0	355.7	49.0	119.7	40.9	38.4
Range of RILs	(1–5)	46.9–576.8	61.8–665.8	11.0–91.6	65.4–142.4	12.8–63.4	5.8–61.6
Heritability (%)	86.5	86.8	86.6	80.9	49.8	91.3	84.7
**Pooled environments (Heat-stress environments, 2013 and 2014)**							
ICC 4567 (heat sensitive)	2	201.6	278.1	37.5	134.8	28.6	26.1
ICC 15614 (heat tolerant)	5	453.6	570.3	59.6	116.4	51.2	48.7
Contrast analysis between parents	−0.6 *	−252 *	−292.2 *	−22 *	18.4 ^ns^	−22.6 *	−22.6 *
Mean of RILs	3.0	296.0	388.5	53.0	117.2	45.8	37.9
Range of RILs	(1–5)	42.2–516	54.9–672.5	9.01–82.3	37.14–157.5	24.13–58.8	2.61–63.9
Heritability (%)	72.2	81.6	82.3	73.1	19.2	NA	81.6

* significant at *p* = 0.05, ns = Not significant, NA = Not available.

**Table 2:** Correlation among the different traits evaluated in RIL population in two heat-stress environments, non-stress environment and pooled over years.

Environments	Traits	VS	FPod	TS	BM	HI	%PodSet	GY
HSE-2013	VS	1						
HSE-2014	VS	1						
Pooled years	VS	1						
HSE-2013	FPod	0.68 **	1					
HSE-2014	FPod	0.78 **	1					
Pooled years	FPod	0.80 **	1					
HSE-2013	TS	0.67 **	0.97 **	1				
HSE-2014	TS	0.78 **	0.96 **	1				
Pooled years	TS	0.79 **	0.97 **	1				
HSE-2013	BM	0.69 **	0.70 **	0.68 **	1			
HSE-2014	BM	0.15 **	0.40 **	0.38 **	1			
Pooled years	BM	0.61 **	0.67 **	0.65 **	1			
HSE-2013	HI	−0.04 ^ns^	0.22 **	0.25 **	−0.35 **	1		
HSE-2014	HI	0.83 **	0.84 **	0.84 **	0.08 ^ns^	1		
Pooled years	HI	0.62 **	0.70 **	0.72 **	0.24 **	1		
HSE-2013	%PodSet	0.63 **	0.72 **	0.73 **	0.62 **	0.00	1	
HSE-2014	%PodSet	0.61 **	0.59 **	0.60 **	0.05 **	0.62 **	1	
Pooled years	%PodSet	0.71 **	0.77 **	0.78 **	0.50 **	0.59 **	1	
HSE-2013	GY	0.66 **	0.88 **	0.89 **	0.74 **	0.32 **	0.63 **	1
HSE-2014	GY	0.73 **	0.90 **	0.89 **	0.57 **	0.84 **	0.50 **	1
Pooled years	GY	0.79 **	0.89 **	0.88 **	0.78 **	0.76 **	0.69 **	1
	Traits	FPod	TS	BM	HI	%PodSet	GY	
NSE-2013	FPod	1						
NSE-2013	TS	0.94 **	1					
NSE-2013	BM	0.60 **	0.63 **	1				
NSE-2013	HI	0.15 **	0.22 **	−0.07 ^ns^	1			
NSE-2013	%PodSet	0.23 **	0.27 **	0.17 **	0.05 ^ns^	1		
NSE-2013	GY	0.63 **	0.69 **	0.91 **	0.33 **	0.17 **	1	

** Significant at *p* < 0.01, respectively. ns: Non-significant. HSE-2013: Heat-stress environment—2013; HSE-2014: Heat-stress environment-2014; NSE-2013: Non-stress environment-2013; Pooled years: Pooled over HSE-2013 and HSE-2014; VS, Visual Score; FPod, Number of Filled Pods per Plot; TS, Total Number of Seeds Per Plot; BM, Biomass; HI, Harvest Index; %PodSet, Percentage Pod Setting; GY, Grain Yield per Plot.

**Table 3:** Identification of QTLs associated with heat tolerance in ICC 4567 × ICC 15614 derived RIL population.

LG	Marker Interval	Trait	QTL Name	Heat-Stress Environment, 2013				Heat-Stress Environment, 2014				Pooled Environments			
				Position (cM)	%PVE	LOD	Add	Position (cM)	%PVE	LOD	Add	Position (cM)	%PVE	LOD	Add
CaLG05	Ca5_44667768-Ca5_46955940	FPod	*qfpod02_5*	4.41	11.57	8.37	27.93	5.41	12.03	7.79	27.31	5.41	12.03	9.41	28.83
		TS	*qts02_5*	5.41	12.00	8.54	36.14	5.41	10.00	7.30	31.27	5.41	10.00	9.07	35.27
		GY	*qgy02_5*	4.41	16.04	11.69	4.72	4.41	16.56	12.00	4.61	4.41	16.56	13.17	4.64
		%PodSet	*q%podset06_5*	6.41	11.51	8.04	3.47	6.41	13.30	9.20	3.40	6.41	13.30	9.48	3.47
CaLG06	Ca6_7846335-Ca6_14353624	VS	*qvs05_6*	62.41	11.07	9.79	0.05	61.51	9.04	7.26	0.06	61.51	9.04	9.54	0.06
		FPod	*qfpod03_6*	62.41	6.56	5.10	20.88	63.40	5.92	4.10	19.01	62.41	5.92	5.22	19.91
		GY	*qgy03_6*	62.41	4.43	3.68	2.48	62.41	3.92	3.21	2.24	62.41	3.92	3.58	2.24
		%PodSet	*q%podset08_6*	63.41	8.44	6.22	3.00	65.41	6.96	4.61	2.46	64.41	6.96	5.97	2.77

VS, Visual Score; FPod, Number of Filled Pods per Plot; TS, Total Number of Seeds per Plot; %PodSet, Percentage Pod Setting; GY, Grain Yield per Plot; %PVE, Percentage of Phenotypic Variance Explained; Add, additive effect, where a positive value indicates that ICC 15614 allele was favorable, and a negative value ICC 4567 allele was favorable; LOD, likelihood of Odds Ratio; LG, Linkage Group.

**Table 4:** Epistatic effect, and epistatic × environment interaction QTL found in RIL population (ICC 4567 × ICC 15614) in two heat-stress environments, 2013 and 2014.

SL. No.	Trait	QTL_i	LG	Marker Interval (QTL i)	Position (QTL_i)	QTL_j	LG	Marker Interval (QTL j)	Position (QTL_j)	AA	h^2^ (%) (AA)	h^2^ (%) (AAE)
1	VS	*eqvs1_1*	1	Ca1_1732919Ca1_4429044	48.5	*eqvs4_7*	7	Ca7_3634430-Ca7_6584610	4.6	−0.02 ***	1.02	0.12
2	VS	*neqvs2_4*	4	Ca4_48498166-Ca4_48498181	2.6	*neqvs3_5*	5	Ca5_29367250-Ca5_28166322	30.4	0.03 ***	2.41	0.17
3	FPod	*eqfpod1_2*	2	Ca2_24709295-Ca2_30876552	30.7	*eqfpod2_2*	2	Ca2_34481663-Ca2_35860429	64.8	−8.85 ***	0.73	0.01
4	FPod	*neqfpod3_4*	4	Ca4_48497765-Ca4_48458381	2.2	*neqfpod4_5/neqts9_5*	5	SCAF9_6963365-Ca5_31125913	44.5	13.10 ***	2.21	0.01
5	TS	*eqts1_1*	1	Ca1_11321839-Ca1_11411540	10.8	*eqts11_6*	6	Ca6_51157939-Ca6_23023346	27.8	13.15 ***	0.42	0.01
6	TS	*eqts2_1/eqpodset2_1*	1	Ca1_39746426-Ca1_34727065	26.4	*eqts14_8*	8	Ca8_14753681-Ca8_14587797	5.6	9.78 ***	0.46	0.02
7	TS	*eqts4_2*	2	Ca2_34481663-Ca2_35860429	65.8	*eqts12_6*	6	Ca6_12582861-Ca6_7846335	62.4	−9.79 ***	0.38	0.05
8	TS	*eqts4_2*	2	Ca2_34481663-Ca2_35860429	65.8	*eqts14_8*	8	Ca8_14753681-Ca8_14587797	5.6	16.97 ***	0.96	0.01
9	TS	*eqts7_5*	5	Ca5_45745864-Ca5_44760469	2	*eqts13_6*	6	Ca6_2549991-Ca6_1815278	93.8	−8.86 ***	0.6	0.00
10	TS	*eqts2_1/eqpodset2_1*	1	Ca1_39746426-Ca1_34727065	26.4	*neqts10_6*	6	Ca6_58897252-Ca6_29163667	14.4	17.68 ***	2.22	0.03
11	TS	*neqts3_2*	2	Ca2_32483185-Ca2_32979328	47.7	*neqts6_4*	4	Ca4_47243660-Ca4_44753224	22.3	13.47 ***	2.12	0.01
12	TS	*neqts5_4*	4	Ca4_48458381-Ca4_48475589	2.2	*neqts8_5*	5	Ca5_27604363-Ca5_27361668	35.7	10.76 ***	2.52	0.03
13	TS	*neqts5_4*	4	Ca4_48458381-Ca4_48475589	2.2	*neqts9_5/neqfpod4_5*	5	SCAF9_6963365-Ca5_31125913	44.5	12.02 ***	2.7	0.00
14	GY	*eqgy1_1*	1	Ca1_1732919-Ca1_4429044	45.5	*eqgy2_2*	2	Ca2_34481663-Ca2_35860429	63.8	1.41 ***	0.83	0.01
15	BM	*aaeqbm1_1*	1	Ca1_11685790-Ca1_11372972	9.1	*neqbm2_3*	3	Ca3_24194574-Ca3_22539683	52.9	−2.09 ***	1.22	0.21
16	%PodSet	*eqpodset1_1*	1	Ca1_11685790-Ca1_11372972	10.1	*eqpodset6_4*	4	Ca4_13699195-Ca4_7818876	75.6	−1.33 ***	0.83	0.01
17	%PodSet	*eqpodset2_1/eqts2_1*	1	Ca1_39746426-Ca1_34727065	26.4	*eqpodset6_4*	4	Ca4_13699195-Ca4_7818876	75.6	1.89 ***	0.99	0.03
18	%PodSet	*eqpodset1_1*	1	Ca1_11685790-Ca1_11372972	10.1	*neqpodset4_4*	4	Ca4_48478303-Ca4_48475461	2.5	−1.38 ***	2.13	0.02
19	%PodSet	*neqpodset3_3*	3	Ca3_9400875-SCAF14_6484051	63.2	*neqpodset5_4*	4	Ca4_48269138-Ca4_47243656	11	−1.44 ***	1.84	0.00

VS, Visual Score; FPod, Number of Filled Pods per Plot; TS, Total Number of Seeds per Plot; GY, Grain Yield per Plot; BM, Biomass; %PodSet, Percentage Pod Setting. QTL_i and QTL_j, the two QTL/non-QTL involved in epistatic interaction; AA, additive × additive effect interactions; AAE, epistatic × environment effect interactions, h^2^ (AA): the contribution rate of additive x additive effect interactions; h^2^ (AAE): the contribution rate of epistatic × environment effect interactions. *** Significant at the 0.001 probability level. The underlined QTLs denotes those with an additive effect. *eqpodset2_1/eqts2_1* or *eqts2_1/eqpodset2_1* and *neqts9_5/neqfpod4_5* or *neqfpod4_5/neqts9_5* indicates co-localized loci.

## Supplementary Materials

The following are available online at http://www.mdpi.com/1422-0067/19/8/2166/s1. Figure S1: Intra-specific genetic map of chickpea RIL population (ICC 4567 × ICC 15614) with 271 GBS-based SNPs covering 529.11 cM. Genetic distances (cM) were shown on the left side and the markers were shown on the right side of the bars. The map was constructed using JoinMap 4.1 and Kosambi function, Figure S2: The epistatic QTLs on linkage groups detected by QTLNetwork v 2.0 in the RIL population (ICC 4567 × ICC 15614). Lines joining two QTLs represents the epistatic interaction between them, Figure S3: Relationship of visual score on podding behaviour (VS), Number of Filled Pods per Plot (FPod), Total Number of Seeds per Plot (TS), Biomass (BM), Harvest Index (HI) and Percent Pod Setting (%PodSet) with Grain Yield per Plot (GY) (**a**) during heat-stress environment of 2013 (**b**) during heat-stress environment of 2014 (**c**) of pooled environments (heat-stressed environments, 2013 and 2014) (**d**) during non-stress environment of 2013 (Due to non-availability of VS data, no relationship of VS with GY is presented in non-stress environment, 2013). *X*-axis represents yield components traits e.g., VS, FPod, TS, BM HI and %PodSet; *Y*-axis represents GY; (No. of RILs-292), Figure S4: Likelihood of odds ratio (LOD) curves obtained by composite interval mapping for quantitative trait loci (QTL) mapped for the traits-visual score on podding behaviour (VS), Number of Filled Pods per Plot(FPod), Total Number of Seeds per Plot (TS), Grain Yield per Plot (GY), Biomass (BM), Harvest Index (HI), and Percent Pod Setting (%PodSet) in RIL population (ICC 4567 × ICC 15614) (**a**) in the heat-stress environment-2013 (**b**) in the heat-stress environment-2014 (**c**) in the pooled environments (heat-stress environments, 2013, and 2014) (**d**) in the non-stress environment-2013 (Due to non-availability of VS data, VS was not mapped in non-stress environment, 2013). The vertical lines indicating the threshold LOD value (2.5) determining significant QTL, Figure S5: Pipeline of Bioinformatics analysis: GBS data processing and SNP calling, Table S1: (**a**) Summary sequence data generated genotyping-on 292 RILs and two parents (ICC 4567 and ICC 15614) using GBS approach; (**b**) Summary of called SNPs on 292 RILs and two parents (ICC 4567 and ICC 15614) using GBS approach, Table S2: Features of intra-specific genetic map developed using 271 SNPs and RIL population ICC 4567 × ICC 15614, Table S3: Summary of QTLs identified in two heat-stress environments, pooled environments and non-stressed environment in RIL population (ICC 4567 × ICC 15614), Table S4: Consistent QTLs found across heat-stress environments (2013 and 2014) in RIL population (ICC 4567 × ICC 15614), Table S5: Gene ontology classification for CaLG05, Table S6: Gene ontology classification for CaLG06, Table S7: Gene ontology categorization of 236 genes identified on the genomic region flanked by markers Ca5_44667768-Ca5_46955940 on CaLG05, Table S8: Gene ontology categorization of 550 genes identified on the genomic region flanked by markers Ca6_7846335-Ca6_14353624 on CaLG06, Table S9: (**a**) List of putative candidate genes found to be associated with heat stress on CaLG05 in chickpea, (**b**) List of putative candidate genes found to be associated with heat stress on CaLG06 in chickpea.

## Abbreviations

%PodSet	Pod Setting Percentage
ANOVA	Analysis of Variance
BLUP	Best Linear Unbiased Prediction
BM	Biomass
CaLG	*Cicer arietinum* Linkage Group
CIM	Composite Interval Mapping
cM	Centimorgan
FPod	Number of Filled Pods Per Plot
GY	Grain Yield Per Plot
HI	Harvest Index
ICRISAT	International Crops Research Institute for the Semi-Arid Tropics
LG	Linkage Group
QTL	Quantitative Trait Loci
ReML	Residual Maximum Likelihood
RIL	Recombinant Inbred Line
TS	Total Number of Seeds Per Plot
VS	Visual Scoring
