# Supplementary material for: Molecular Mapping of QTLs for Heat Tolerance in Chickpea
Source: Int J Mol Sci. 2018 Jul 25;19(8):2166. doi: 10.3390/ijms19082166 (PMC6121679; doi:10.3390/ijms19082166)
Supplement: Supplementary file 1 [file ijms-19-02166-s001.zip › ijms-324599-SI/Supplementary Figure 3b.pptx]

## Slide 1
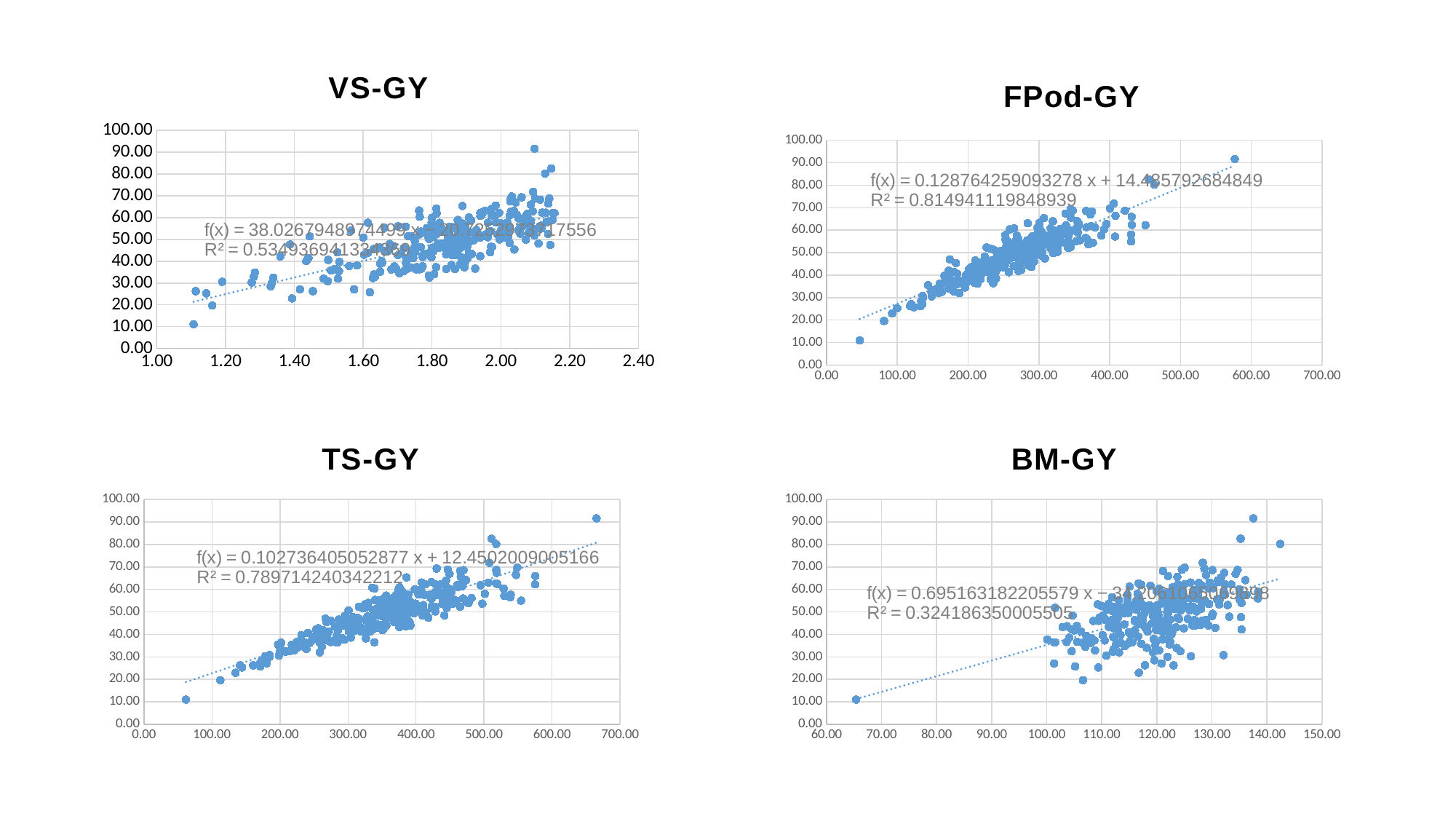

### Chart: VS-GY
| Category | GY |
|---|---|
### Chart: FPod-GY
| Category | GY |
|---|---|
### Chart: TS-GY
| Category | GY |
|---|---|
### Chart: BM-GY
| Category | GY |
|---|---|

## Slide 2
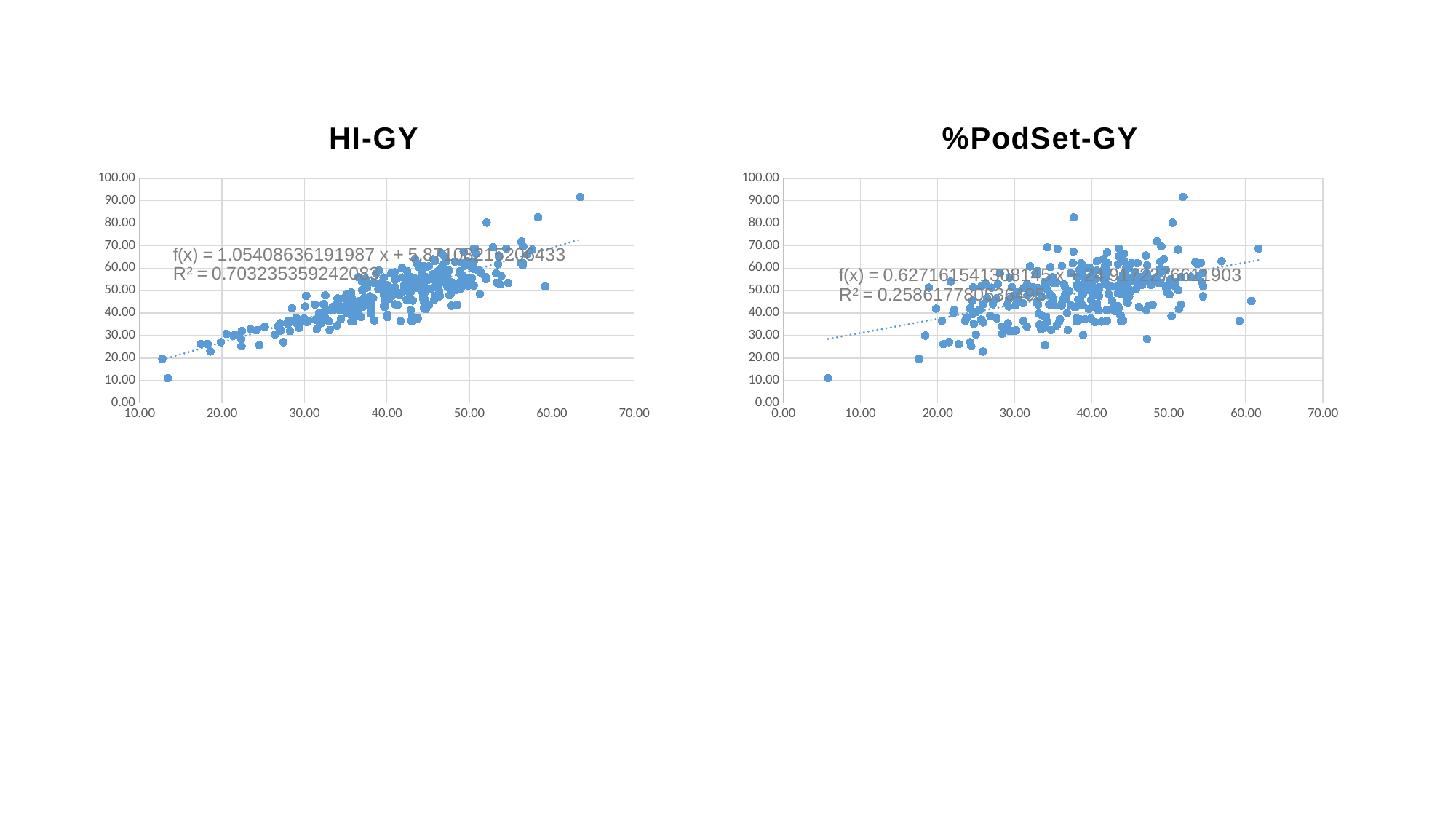

### Chart: HI-GY
| Category | GY |
|---|---|
### Chart: %PodSet-GY
| Category | GY |
|---|---|
