# Supplementary material for: Molecular Mapping of QTLs for Heat Tolerance in Chickpea
Source: Int J Mol Sci. 2018 Jul 25;19(8):2166. doi: 10.3390/ijms19082166 (PMC6121679; doi:10.3390/ijms19082166)
Supplement: Supplementary file 1 [file ijms-19-02166-s001.zip › ijms-324599-SI/Supplementary table 9a & 9b.docx]

| **Supplementary Table 9a. List of putative candidate genes found to be associated with heat stress on CaLG05 in chickpea** | | | | | |
| --- | --- | --- | --- | --- | --- |
| **Gene id** | **Gene name** | **Protein name** | **Function** | **Plant/ Crop** | **Reference** |
| **Ca_04038** | HSP21 | Small heat shock protein, chloroplastic | Have a catalytic role in recovery and protection during heat stress. | *Pisum sativum* (Garden pea) | Chen et al., (1990) |
| **Ca_04067** | F20M13.140 | (Farnesylated protein 6) (AtFP6) | This heat acclimation regulation gene may involve in facilitation of the survival and growth of plants under severe heat-stress conditions | *Arabidopsis thaliana* | Lim et al., (2006) |
| **Ca_04147** | ERF114 | Ethylene-responsive transcription factor ERF114 | May be involved in the regulation of gene expression by stress factors; Helps in heat acclimation and subsequent recovery from heat injury. | *Arabidopsis thaliana* | Müller & Munné-Bosch, (2015),Chen et al., (2015), Larkindale & Huang, (2005),Larkindale & Knight, (2002) |
| **Ca_03948** | CRF4/ ERF066 | Ethylene-responsive transcription factor CRF4 | May be involved in the regulation of gene expression by stress factors; Helps in heat acclimation and subsequent recovery from heat injury. | *Arabidopsis thaliana* | Müller & Munné-Bosch, (2015), Chen et al., (2015), Larkindale & Huang, (2005), Larkindale & Knight, (2002) |
| **Ca_03919** | MYB44 | Transcription factor MYB44 | May have role in abscisic acid signaling pathway; response to abscisic acid, ethylene, salicylic acid. | *Arabidopsis thaliana* | Larkindale & Knight, (2002),Jung et al., (2008; 2014, 2013) |
| **Ca_04058** | TTG1 | Protein TRANSPARENT TESTA GLABRA 1 | Involved in response in ethylene which is associated with heat stress-induced oxidative damage | *Arabidopsis thaliana* | Larkindale & Huang, (2005; 2002) |
| **Ca_03952** | WRKY53 | Probable WRKY transcription factor 53 | Believed to act as convergence node between senescence and biotic and abiotic stress responses, thus appears to contribute to reproductive success. | *Arabidopsis thaliana* | Xie et al., (2014). Zentgraf et al., (2010) |
| **Ca_04125** | PER52 | Peroxidase 52 | Involved in responses to environmental responses such as wound and oxidative stress. | *Arabidopsis thaliana* | Veitch, (2004) |
| **Ca_03916** | SKP2B | F-box protein SKP2B | Involved in heat acclimation and abiotic responses | *Arabidopsis thaliana* | Gupta et al., (2015) |
| **Ca_04100** | WRKY70 | Probable WRKY transcription factor 70 | Involved in negative regulation of leaf senescence and defense signal pathways | *Arabidopsis thaliana* | Besseau et al., (2012),Ülker et al., (2007) |
| **Ca_03974** | COL5 | Zinc finger protein CONSTANS-LIKE 5 | Involved in oxidative stress and regulation of flower development. | *Arabidopsis thaliana* | Rizhsky et al., (2004) |

| **Supplementary Table 9b. List of putative candidate genes found to be associated with heat stress on CaLG06 in chickpea** | | | | | |
| --- | --- | --- | --- | --- | --- |
| **Gene id** | **Gene name** | **Protein name** | **Function** | **Plant/ Crop** | **Reference** |
| **Ca_08536** | HSFA5 | Heat stress transcription factor A-5 | May have role in heat stress response | *Arabidopsis thaliana* | From UniProt database  http://www.uniprot.org/uniprot/Q94BZ5 |
| **Ca_08627** | HSFA4C/HSF20/ RHA1 | Heat stress transcription factor A-4c | Acts as a possible factor in high temperature response | *Arabidopsis thaliana* | From UniProt database  http://www.uniprot.org/uniprot/Q9FK72 |
| **Ca_09569** | ANNAT7 / ANN7 | Annexin D7 (AnnAt7) | A possible role in heat stress response | *Arabidopsis thaliana* | Cantero et al., (2006) |
| **Ca_08522** | FKBP65/ ROF2 | Peptidyl-prolyl cis-trans isomerase FKBP65 | This heat stress protein found to be involved in long term acquired tthermotolerance modulating heat shock transcription factor HsfA2 and interacting with HSP90.1. | *Arabidopsis thaliana* | Meiri et al., (2010) |
| **Ca_08619** | HSP26-A | Heat shock protein 26A | May involved in cellular response to stress | *Glycine max* | Czarnecka et al., (1988) |
| **Ca_05050** | CPN60-2 | Chaperonin CPN60-2/HSP60 | Involved in pro-apoptotic role in plants under drastic heat shock | *Arabidopsis thaliana* | Rikhvanov et al., (2007) |
| **Ca_09578** | ERF060 | Ethylene-responsive transcription factor ERF060 | May be associated with the regulation of gene expression by stress factors; May play a role in to control the floral organ senescence and abscission. | *Arabidopsis thaliana* | Chen et al., (2015; 2005; 2015; 2002) |
| **Ca_08582** | RAP2-11/ ERF002 | Ethylene-responsive transcription factor RAP2-11 | May be associated with the regulation of gene expression by stress factors; May play a role in to control the floral organ senescence and abscission. | *Arabidopsis thaliana* | Chen et al., (2015; 2005; 2015; 2002) |
| **Ca_09594** | HIPP26 / FP6 | Heavy metal-associated isoprenylated plant protein 26 | May have direct role in heat acclimation | *Arabidopsis thaliana* | Lim et al., (2006) |
| **Ca_25056** | - | Universal stress protein A-like protein | Involved in response to stress | *Arabidopsis thaliana* | Kim et al., (2015) |
| **Ca_08560** | THI1 | Thiamine thiazole synthase | May play roles in adaptation to various stress conditions and in DNA damage tolerance | *Citrus sinensis* | Ribeiro et al., (2005),Machado et al., (1997) |
| **Ca_08509** | AHK3/ ORE12 | Histidine kinase 3 | Involved in cellular response to abscisic acid; regulation of flower development | *Arabidopsis thaliana* | Desikan et al., (2006),Alonso et al., (1999) |
| **Ca_05133** | RAN1/ HMA7 | Copper-transporting ATPase RAN1 | Associated with response to ethylene, stress signaling and regulation of stomatal movement | *Arabidopsis thaliana* | Desikan et al., (2006),Alonso et al., (1999) |
| **Ca_05076** | FER / AAK1 /SIR / SRN | Receptor-like protein kinase FERONIA | Helps in mediating male – female interaction during pollen tube reception and thus play important role in reproduction; involved in plant growth process through cell elongation. | *Arabidopsis thaliana* | Guo et al., (2009),Escobar-Restrepo et al., (2007) |

Alonso JM, Hirayama T, Roman G, Nourizadeh S, Ecker JR (1999) EIN2, a bifunctional transducer of ethylene and stress responses in *Arabidopsis*. Science 284 :2148–2152

Aviezer-Hagai K, Skovorodnikova J, Galigniana M, Farchi-Pisanty O, Maayan E, Bocovza S, Efrat Y, von Koskull-Dӧring P, Ohad N, Breiman A (2007) *Arabidopsis* immunophilins ROF1 (AtFKBP62) and ROF2 (AtFKBP65) exhibit tissue specificity, are heat-stress induced, and bind HSP90. Plant Mol Biol 63: 237–255

Besseau S, Li J, Palva ET (2012) WRKY54 and WRKY70 co-operate as negative regulators of leaf senescence in *Arabidopsis thaliana*. J Exp Bot 63:2667–2679

Cantero A, Barthakur S, Bushart T, Chou S, Morgan R, Fernandez M, Clark G, Roux S (2006) Expression profiling of the Arabidopsis annexin gene family during germination, de-etiolation and abiotic stress. Plant Physiol Bioch 44:13–24

Chen Q, Lauzon LM, DeRocher AE, Vierling E (1990) Accumulation, stability, and localization of a major chloroplast heat-shock protein. J Cell Biol 110:1873–83

Chen WH, Li PF, Chen MK, Lee YI, Yang CH (2015) FOREVER YOUNG FLOWER Negatively Regulates Ethylene Response DNA-binding Factors (EDFs), by Activating an Ethylene-Responsive Factor (ERF), to Control Arabidopsis Floral Organ Senescence and Abscission. Plant Physiol pp–00433

Czarnecka E, Nagao R, Key J, Gurley W (1988) Characterization of Gmhsp26-A, a stress gene encoding a divergent heat shock protein of soybean: heavy-metal-induced inhibition of intron processing. Mol Cell Biol 8:1113–1122

Desikan R, Last K, Harrett-Williams R, Tagliavia C, Harter K, Hooley R, Hancock JT, Neill SJ (2006) Ethylene-induced stomatal closure in Arabidopsis occurs via AtrbohF-mediated hydrogen peroxide synthesis. Plant J 47: 907–916

Escobar-Restrepo JM, Huck N, Kessler S, Gagliardini V, Gheyselinck J, Yang WC, Grossniklaus U (2007) The FERONIA receptor-like kinase mediates male-female interactions during pollen tube reception. Science 317:656–660

Guo H, Li L, Ye H, Yu X, Algreen A, Yin Y (2009) Three related receptor-like kinases are required for optimal cell elongation in *Arabidopsis thaliana*. Proceedings of the National Academy of Sciences 106:7648–7653

Gupta S, Garg V, Kant C, Bhatia S (2015) Genome-wide survey and expression analysis of Fbox genes in chickpea. BMC Genomics 16: 67

Jung C, Seo JS, Han SW, Koo YJ, Kim CH, Song SI, Nahm BH, Do Choi Y, Cheong J-J (2008) Overexpression of AtMYB44 enhances stomatal closure to confer abiotic stress tolerance in transgenic *Arabidopsis*. Plant Physiol 146: 623–635

Kim DJ, Bitto E, Bingman CA, Kim HJ, Han BW, Phillips GN (2015) Crystal structure of the protein At3g01520, a eukaryotic universal stress protein-like protein from *Arabidopsis thaliana i*n complex with AMP. Proteins: Struct Funct Bioinf 83(7) 1368-1373

Larkindale J, Huang B (2005) Effects of abscisic acid, salicylic acid, ethylene and hydrogen peroxide in thermtolerance and recovery for creeping bent grass. Plant Growth Regul 471:17–28

Larkindale J, Knight MR (2002) Protection against heat stress-induced oxidative damage in *Arabidopsis* involves calcium, abscisic acid, ethylene, and salicylic acid. Plant Physiol 128:682–695

Lim CJ, Yang KA, Hong JK, Choi JS, Yun DJ, Hong JC, Chung WS, Lee SY, Cho MJ, Lim CO (2006) Gene expression profiles during heat acclimation in *Arabidopsis thaliana* suspension-culture cells. J Plant Res 119:373–83

Machado CR, Praekelt UM, de Oliveira RC, Barbosa ACC, Byrne KL, Meacock PA, Menck CF (1997) Dual role for the yeast THI4 gene in thiamine biosynthesis and DNA damage tolerance. J Mol Biol 273, 114–121

Meiri D, Tazat K, Cohen-Peer R, Farchi-Pisanty O, Aviezer-Hagai K, Avni A, Breiman A (2010) Involvement of *Arabidopsis* ROF2 (FKBP65) in thermotolerance. Plant Mol Biol 72:191–203

Müller M, Munné-Bosch S (2015) Ethylene response factors: a key regulatory hub in hormone and stress signaling. Plant Physiol 169:32–41

Persak H, Pitzschke A (2014) Dominant repression by Arabidopsis transcription Factor MYB44 causes oxidative damage and hypersensitivity to abiotic stress. Int J Mol Sci 15:2517–2537

Persak H, Pitzschke A (2013) Tight interconnection and multi-level control of Arabidopsis MYB44 in MAPK cascade signalling. PloS one 8 (2): e57547

Ribeiro DT, Farias LP, de Almeida JD, Kashiwabara PM, Ribeiro AFC, Silva-Filho MC, Menck CFM, Van Sluys M-A (2005) Functional characterization of the thi1 promoter region from *Arabidopsis thaliana*. J Exp Bot 56:1797–804

Rikhvanov EG, Gamburg KZ, Varakina NN, Rusaleva TM, Fedoseeva IV, Tauson EL, Stupnikova IV, Stepanov AV, Borovskii GB, Voinikov VK (2007) Nuclear-mitochondrial cross-talk during heat shock in *Arabidopsis* cell culture. Plant J 52:763–778

Rizhsky L, Davletova S, Liang H, Mittler R (2004) The zinc finger protein Zat12 is required for cytosolic ascorbate peroxidase 1 expression during oxidative stress in *Arabidopsis*. J Biol Chem 279:11736–11743

Ülker B, Mukhtar MS, Somssich IE (2007) The WRKY70 transcription factor of Arabidopsis influences both the plant senescence and defense signaling pathways. Planta 226:125–137

Veitch NC (2004) Structural determinants of plant peroxidase function. Phytochem Rev 3:3–18

Xie Y, Huhn K, Brandt R, Potschin M, Bieker S, Straub D, Doll J, Drechsler T, Zentgraf U, Wenkel S (2014) REVOLUTA and WRKY53 connect early and late leaf development in *Arabidopsis*. Development 141:4772–4783

Zentgraf U, Laun T, Miao Y (2010) The complex regulation of WRKY53 during leaf senescence of *Arabidopsis thaliana.*  Eur J Cell Biol 89:133–137
