# Supplementary figures and images for: Molecular Mapping of QTLs for Heat Tolerance in Chickpea
Source: Int J Mol Sci. 2018 Jul 25;19(8):2166. doi: 10.3390/ijms19082166 (PMC6121679; doi:10.3390/ijms19082166)

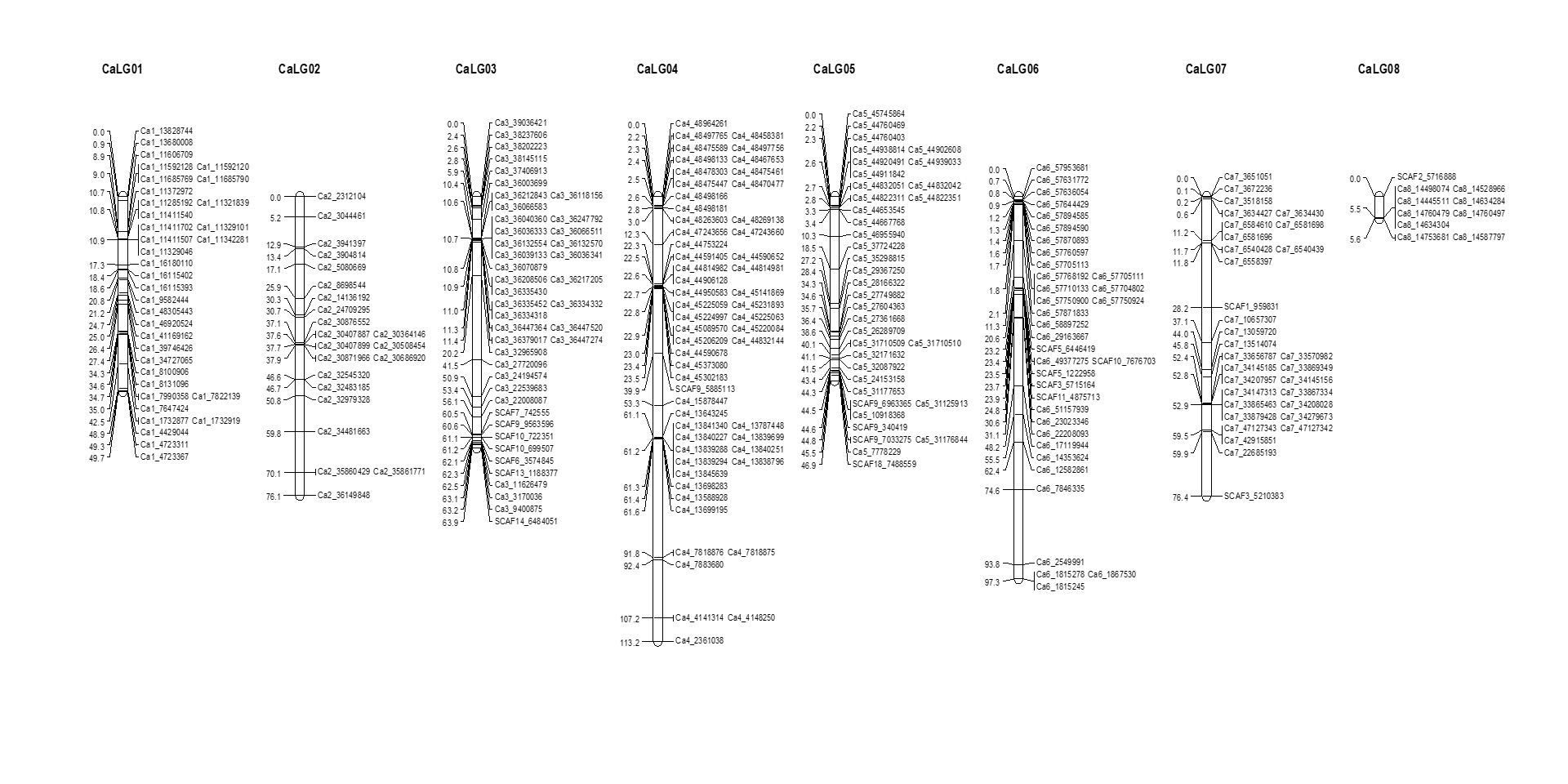

Supplement: Supplementary file 1 [file ijms-19-02166-s001.zip › ijms-324599-SI/Supplementary Figure 1.tif]

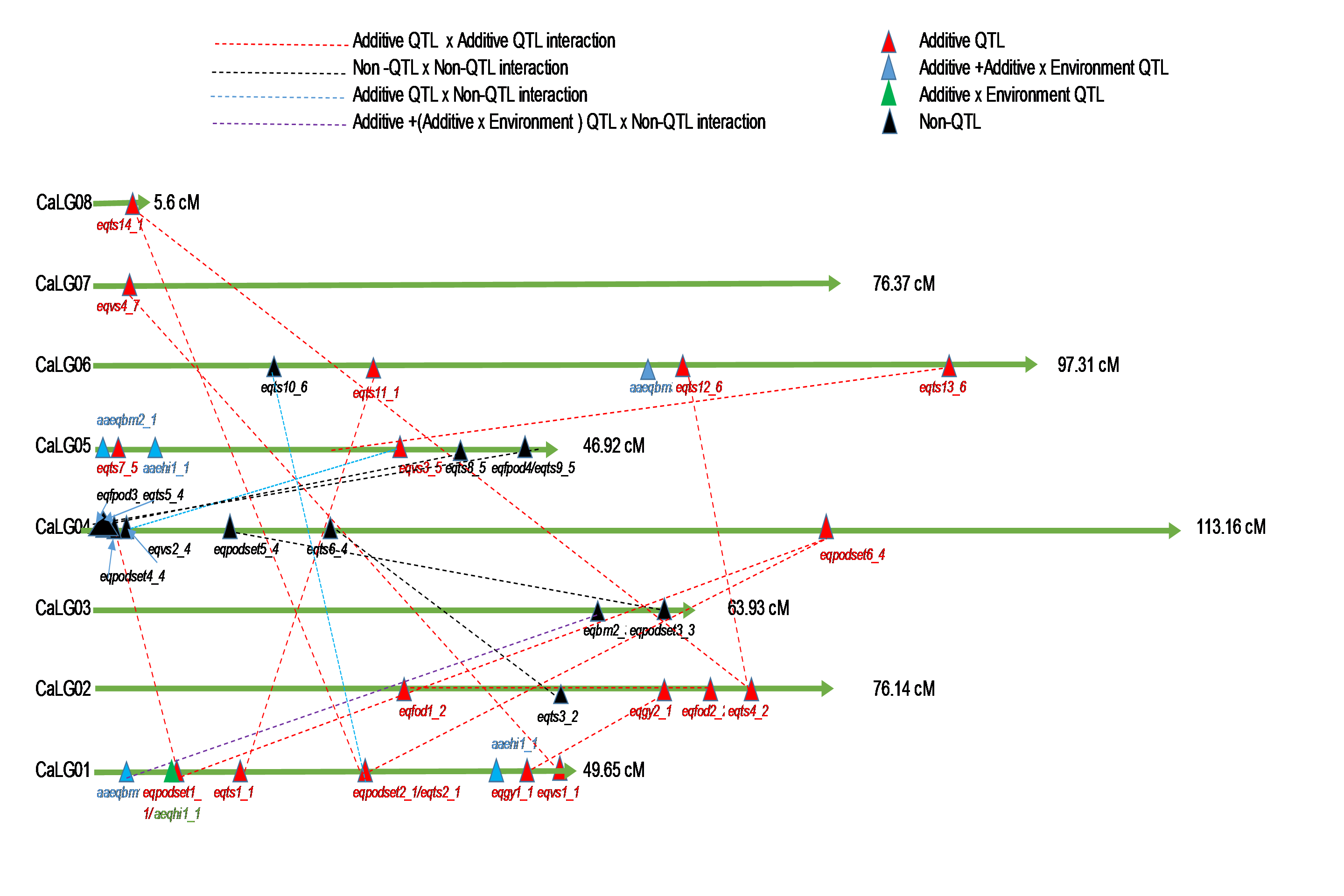

Supplement: Supplementary file 1 [file ijms-19-02166-s001.zip › ijms-324599-SI/Supplementary Figure 2.tif]

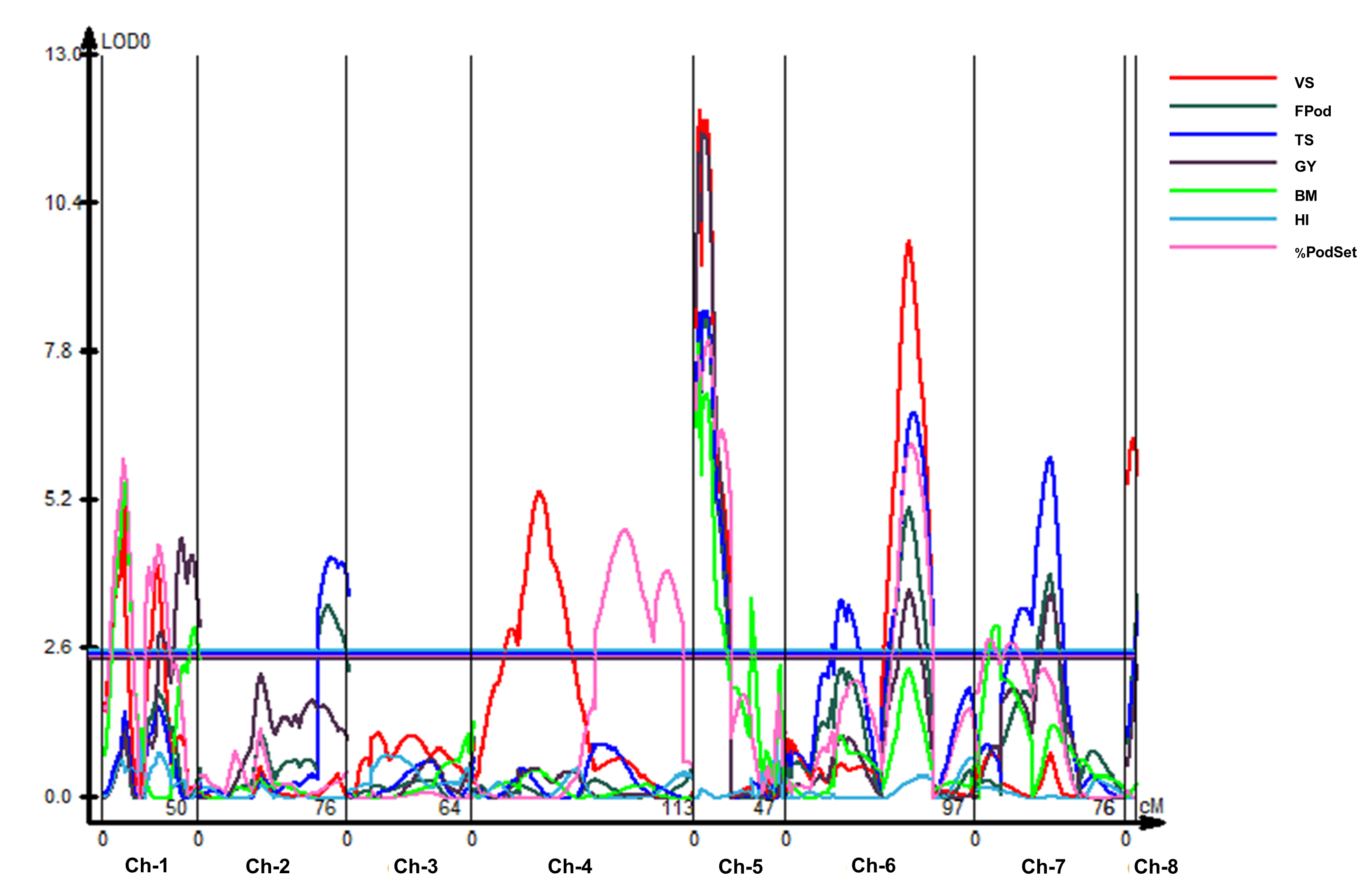

Supplement: Supplementary file 1 [file ijms-19-02166-s001.zip › ijms-324599-SI/Supplementary Figure 4a.tif]

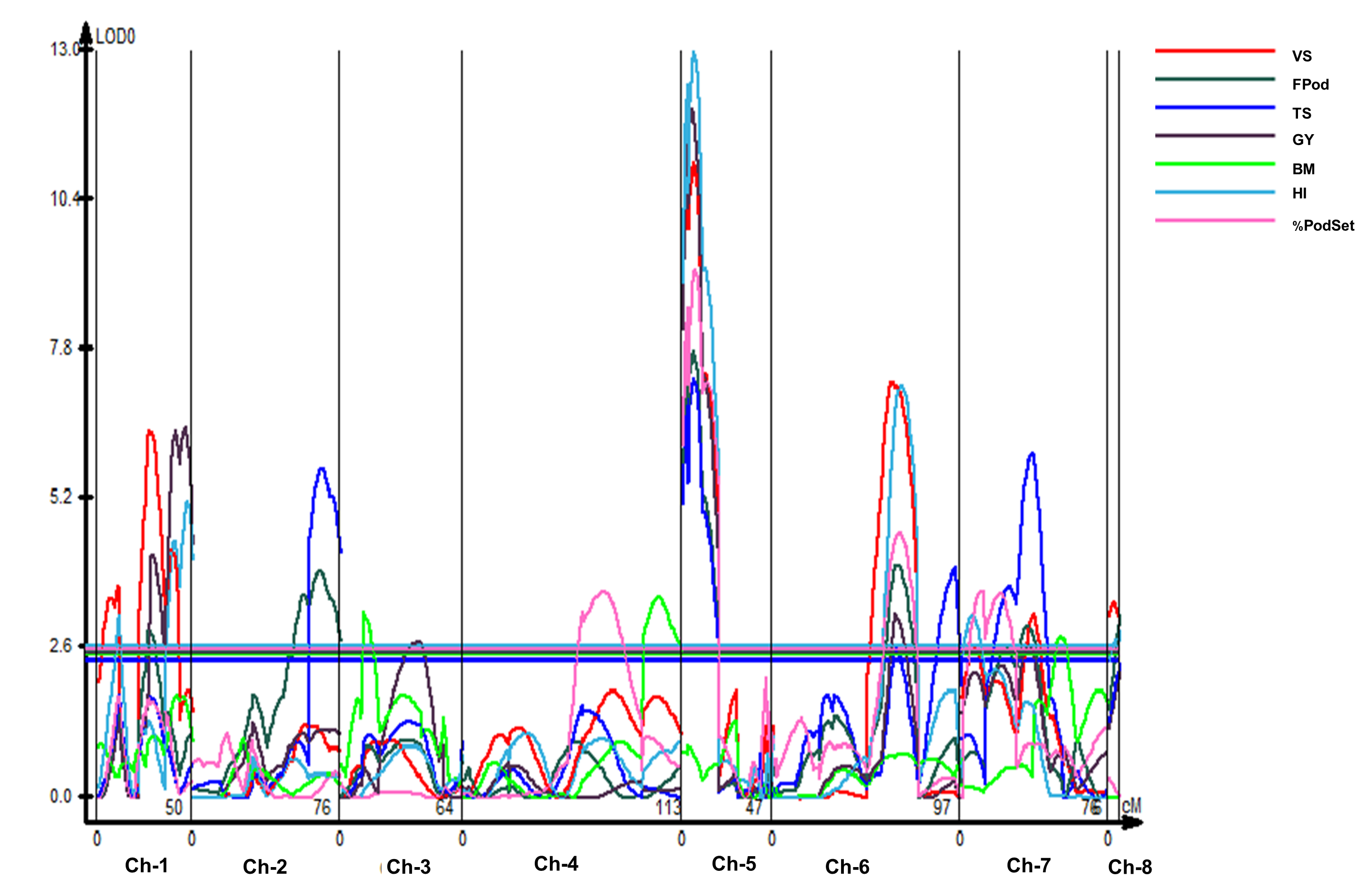

Supplement: Supplementary file 1 [file ijms-19-02166-s001.zip › ijms-324599-SI/Supplementary Figure 4b.tif]

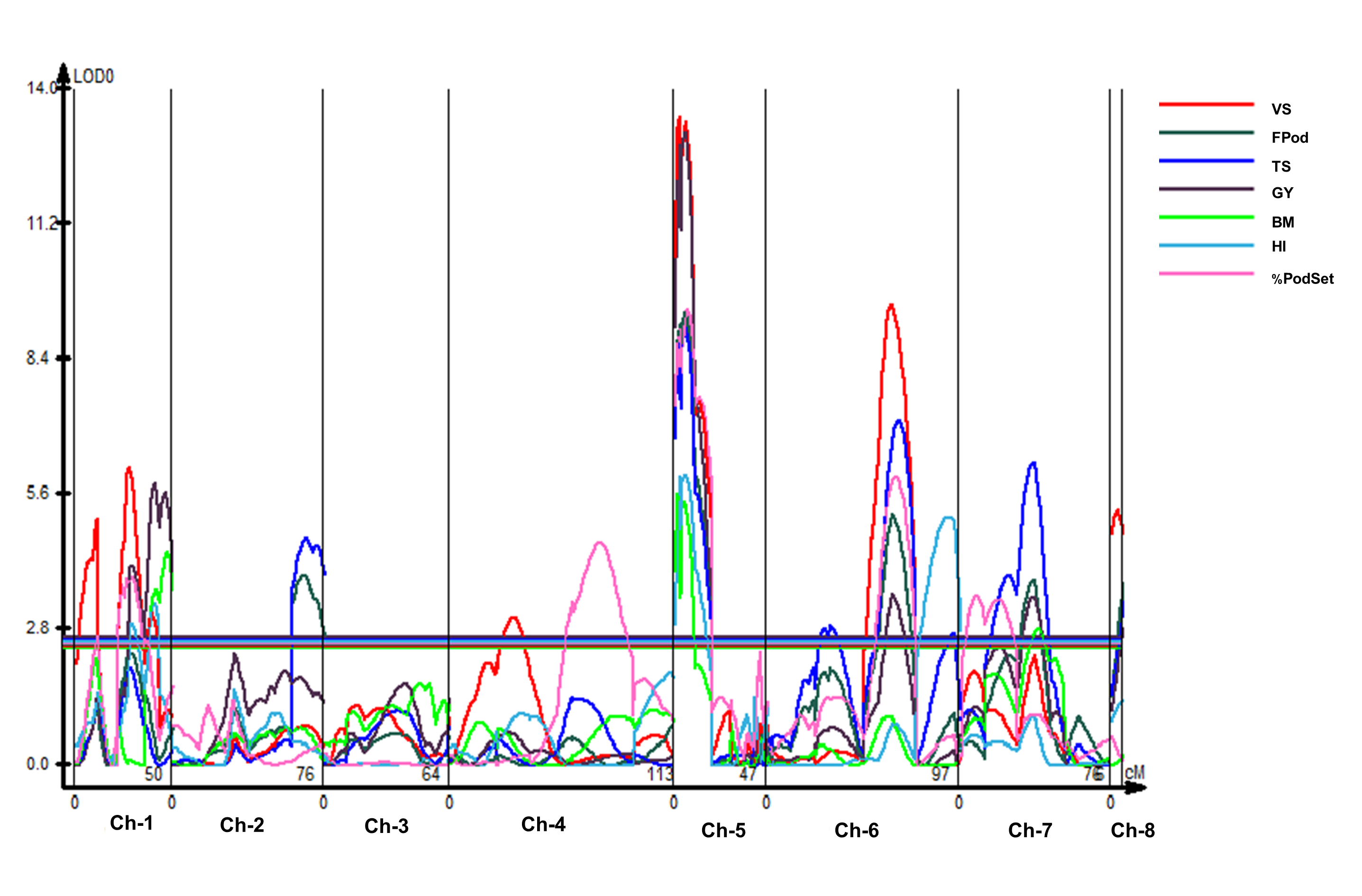

Supplement: Supplementary file 1 [file ijms-19-02166-s001.zip › ijms-324599-SI/Supplementary Figure 4c.tif]

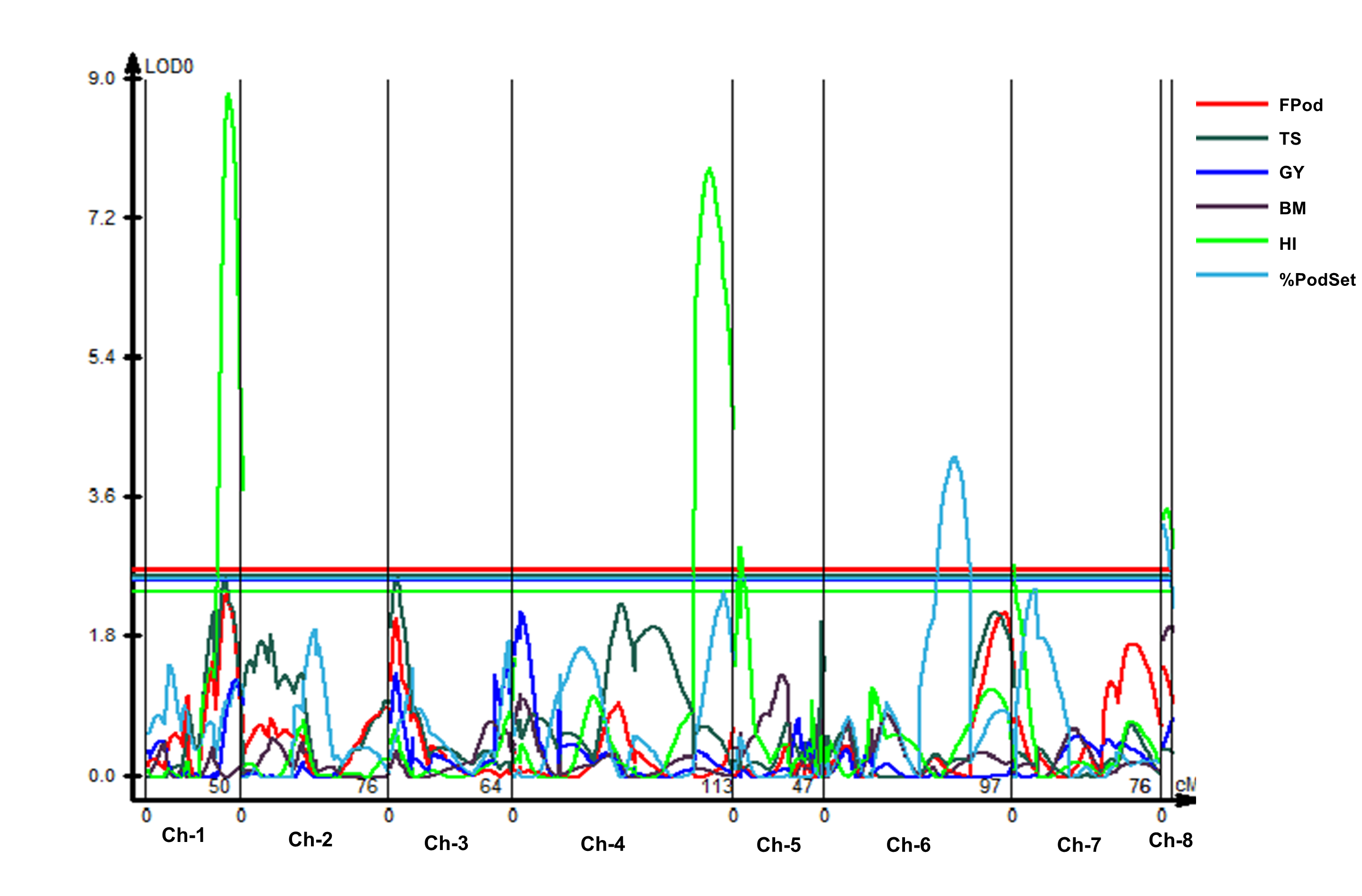

Supplement: Supplementary file 1 [file ijms-19-02166-s001.zip › ijms-324599-SI/Supplementary Figure 4d.tif]
